# Osteocalcin Levels in Male Idiopathic Hypogonadotropic Hypogonadism: Relationship With the Testosterone Secretion and Metabolic Profiles

**DOI:** 10.3389/fendo.2019.00687

**Published:** 2019-10-11

**Authors:** Yu-Ying Yang, Si-Chang Zheng, Wen-Cui Wang, Zu-Wei Yang, Chang Shan, Yu-Wen Zhang, Yan Qi, Yu-Hong Chen, Wei-Qiong Gu, Wei-Qing Wang, Hong-Yan Zhao, Jian-Min Liu, Shou-Yue Sun

**Affiliations:** ^1^Department of Endocrine and Metabolic Diseases, Shanghai Clinical Center for Endocrine and Metabolic Diseases, Shanghai Jiao-Tong University School of Medicine, Shanghai Institute of Endocrine and Metabolic Diseases, Rui-jin Hospital, Shanghai, China; ^2^Department of Endocrine and Metabolic Diseases, Shanghai Jiao-Tong University School of Medicine, Rui-jin Hospital North, Shanghai, China

**Keywords:** osteocalcin, idiopathic hypogonadotropic hypogonadism, gonadotropin, testosterone, metabolism

## Abstract

Idiopathic hypogonadotropic hypogonadism (IHH) patients are characterized by the absence of puberty and varying degrees of deteriorated metabolic conditions. Osteocalcin (OC) could regulate testosterone secretion and energy metabolism, but it remains unknown whether such an effect exists in IHH patients. Our study is aimed to examine the relationship between serum OC levels with testosterone and its responsiveness to gonadotropin stimulation and metabolic profiles in male IHH patients. A total of 99 male patients aged 18–37 years and diagnosed with IHH were enrolled in the current study, and the relationships between OC and testicular volume, baseline total testosterone (TT), free testosterone (FT), and peak TT (Tmax) levels after human chorionic gonadotropin (hCG) stimulation, gonadotropin responsiveness index (GRI), which is calculated by dividing Tmax by testicular volume, as well as metabolic profiles, such as 2-h post-challenge glucose (2hPG) and fat percentage (fat%), were analyzed. The results showed that OC had an independent negative relationship with testicular volume (*r* = −0.253, *P* = 0.012) and a positive association with Tmax (*r* = 0.262, *P* = 0.014) after adjusting for confounders. In addition, OC was a major determinant of GRI (adjusted *R*^2^ for the model = 0.164, *P* = 0.012), fat% (adjusted *R*^2^ for the model = 0.100, *P* = 0.004), and 2hPG (adjusted *R*^2^ for the model = 0.054, *P* = 0.013) in IHH patients. In conclusion, OC is associated with testosterone secretion upon gonadotropin stimulation, glucose metabolism, and fat mass variations in IHH. This study was registered at clinicaltrials.gov (NCT02310074).

## Introduction

Idiopathic hypogonadotropic hypogonadism (IHH) is a rare disease characterized by the absence of puberty with low gonadotropin and sex steroid levels, with a prevalence of 1–10/100000 (1). This condition is pathophysiologically caused by the congenitally deficient secretion of gonadotropin-releasing hormone (GnRH), which stimulates the release of gonadotropins and regulates the development of gonads. Currently, gonadotropin therapy remains the main treatment for IHH patients who demand fertility. However, a subnormal testosterone response to gonadotropin stimulation has been previously demonstrated in IHH (2, 3), and as a result, only 60–70% of patients achieved fertility after gonadotropin treatment (4, 5).

In addition to the defect in sex development, IHH patients have varying degrees of deteriorated metabolic profiles, including higher body mass index (BMI), fasting blood glucose (FBG), triglycerides (TG), total cholesterol (TC), low-density lipoprotein cholesterol (LDL-C), and fat percentage (fat%), as well as lower high-density lipoprotein cholesterol (HDL-C) and fat-free mass compared with healthy controls (6–8), which might be attributed to the low levels of testosterone (9–13) and gonadotropins (8).

Although IHH is a disease of the gonads with dysregulated metabolic profiles, actually, gonads and bone are mutually dependent (14); the cross-talk between skeleton, energy metabolism, and male fertility is a major interest in the field (15, 16). Osteocalcin (OC) is an osteoblast-derived molecular that is synthesized in bone and is traditionally regarded as a marker of bone formation (17). Recently, the extra-skeletal physiological functions of OC have been revealed, including modulation of male fertility and energy metabolism. Evidence from mouse and human studies has demonstrated that OC can regulate male fertility through promoting testosterone biosynthesis by Leydig cells, thus favoring spermatogenesis via the pancreas–bone–testis axis, which is independent of the hypothalamus–pituitary–testicle axis (16, 18). In addition, OC can regulate glucose and energy homeostasis via various mechanisms, such as promoting islet β cell proliferation and insulin secretion (19), improving insulin sensitivity (20), and increasing energy expenditure (21). However, the correlations between OC and the changes in serum testosterone levels and the responsiveness of testicle to gonadotropin in IHH have never been reported. Due to the functional absence of the preeminent hypothalamus–pituitary–testis axis, IHH is a suitable model to study the relationship between OC and reproductive function. Determination of the association between OC and metabolic parameters in this group of patients is also of interest.

## Materials and Methods

### Subjects

Ninety-nine male patients aged 18–37 years and diagnosed with IHH according to the American Association of Clinical Endocrinologists Medical Guidelines (22) were consecutively recruited from the outpatient service of the Department of Endocrine and Metabolic Diseases, Rui-jin Hospital. Patients treated with testosterone, pulsatile GnRH, or gonadotropin therapy in recent 3 months or longer than 6 months were excluded.

The study protocol was approved by the Institutional Review Board of the Rui-jin Hospital in Shanghai, and informed consent was obtained from each participant before the study. This trial was also registered at clinicaltrials.gov (NCT02310074).

### Clinical and Biochemical Measurements

Clinical measurements including height, weight, BMI, fat%, and testicular volume were evaluated. Specifically, height was measured without shoes to the nearest 0.5 cm using a portable stadiometer, and weight with light clothing was measured to the nearest 0.1 kg on a digital scale. BMI was calculated by dividing the weight by height squared. Testicular volume was examined by ultrasound examination of scrotal content (GE LOGIQ E9; GE Healthcare) and calculated by the formula length × width × depth × 0.71 (23), and the mean values of right and left testicular volumes [(right + left)/2] were used for analysis. Fat% was measured using a bioelectrical impedance analyzer (VBODY HBF-358, Omron).

Blood samples were immediately centrifuged after the collection, and the serum was stored at −80°C until assayed. The serum concentrations of OC were measured using commercial kits (Elecsys N-MID osteocalcin, Cobas 601; Roche Diagnostics) according to the manufacturer's instructions. Serum luteinizing hormone (LH), follicle-stimulating hormone (FSH), total testosterone (TT), and free testosterone (FT) were measured by chemiluminescence immunoassays (Abbott). The lowest level of the measurement range was used instead when the TT and FT levels were too low to detect. Measurements of liver and kidney function, including alanine transferase (ALT), aspartate transferase (AST), γ-glutamyl transpeptidase (GGT), alkaline phosphatase (ALP), serum creatinine (Cr), serum uric acid (UA), and serum urea nitrogen (BUN), were examined using an autoanalyzer (Modular E170; Roche). Moreover, TG, TC, HDL-C, and LDL-C concentrations were tested (Modular E170; Roche). Parathyroid hormone (PTH) was measured by an intact immunoradiometric assay (IRMA) (Abbott Diagnostics Division, USA). Serum 25-hydroxyvitamin D (25(OH)D) concentration was measured by an enzyme immunoassay (Immunodiagnostic Systems, UK).

### Oral Glucose Tolerance Test (OGTT) and Human Chorionic Gonadotropin (hCG) Stimulation Tests

All patients received a 75-g OGTT in our center after 10 h of fasting. Fasting plasma glucose (FPG) and 2-h post-challenge plasma glucose (2hPG) concentrations were measured using the glucose oxidase method and an autoanalyzer (Modular P800; Roche) immediately after the blood was drawn.

In addition, all patients underwent a 3-day hCG stimulation test. Patients received an intramuscular injection of 2,000 U hCG at 0800 h, and the venous blood for TT measurements was collected at −15 and 0 min before the injection and 24, 48, and 72 h after the injection. Tmax is defined as the peak TT (Tmax) level during the hCG stimulation test. We used the gonadotropin response index (GRI), which is calculated by dividing Tmax by testicular volume to represent the responsiveness to gonadotropin per volume of the testicle.

### Statistics

The distribution of continuous variables was examined by the Shapiro–Wilks test. The results are presented as the mean ± SD for normally distributed variables or median (interquartile range) for skewed parameters. Group differences were compared with ANOVA tests for normally distributed variables, whereas the non-parametric Mann–Whitney *U*-test was performed for skewed parameters. Bonferroni post-test analysis was used. Spearman's bivariate correlation tests and partial correlation analyses were conducted to study the associations between OC and anthropometric indices, FBG, 2hPG, and serum biochemical parameters, with adjustment for confounders. Multivariate stepwise linear regression was used to test the combined effect of the independent factors on the dependent variable. All statistical tests were two-tailed. A value of *P* < 0.05 was considered significant. The statistical analysis was performed using SPSS 23.0 (SPSS, Inc.).

## Results

### Baseline Clinical Characteristics of IHH Patients by Quartiles of Serum OC Levels

The baseline characteristics of the subjects are presented according to the OC quartiles (Table 1). Age, BMI, fat%, ALT, and testicular volume decreased, while GRI and ALP increased with elevation of OC. LH, FSH, TT, FT, Tmax, AST, GGT, BUN, Cr, UA, TG, TC, HDL-C, LDL-C, FBG, 2hPG, 25(OH)D, and PTH did not differ significantly with respect to the OC quartiles.

**Table 1:** Baseline clinical characteristics of IHH patients by quartiles of serum OC levels.

	**Serum OC levels (ng/ml)**					
	**Overall population**	**Quartile 1**	**Quartile 2**	**Quartile 3**	**Quartile 4**	***P***
	**46 (34–66) *N* = 99**	**28 (23–32) *N* = 25**	**41 (39–44) *N* = 25**	**58 (51–62) *N* = 26**	**81 (74–102) *N* = 23**	
Age (years)	24 (20–27)	27 (24–30)	25 (24–30)	22 (20–25)^a^	19 (19–22)^a^	0.000
BMI (kg/m^2^)	23.08 ± 4.37	24.71 ± 4.74	24.01 ± 4.85	21.80 ± 3.86	21.72 ± 3.20	0.026
Fat%	26.95 ± 5.38	29.58 ± 5.26	28.74 ± 4.04	24.82 ± 5.83^a^	24.78 ± 4.59^a^	0.005
LH (mIU/ml)	0.15 (0.08–0.42)	0.19 (0.09–0.50)	0.17 (0.07–0.55)	0.12 (0.08–0.21)	0.17 (0.08–0.60)	0.701
FSH (mIU/ml)	0.50 (0.33–1.17)	0.56 (0.35–1.12)	0.50 (0.30–1.39)	0.62 (0.38–1.44)	0.47 (0.36–1.36)	0.948
TT (ng/ml)	0.33 (0.24–0.49)	0.39 (0.27–0.56)	0.33 (0.22–0.65)	0.33 (0.25–0.46)	0.29 (0.18–0.42)	0.230
FT (pg/ml)	1.96 (1.50–2.415)	2.17 (1.69–2.50)	1.83 (1.58–2.26)	1.94 (1.57–2.32)	1.82 (1.25–2.36)	0.409
Tmax (ng/ml)	1.02 (0.76–1.93)	1.04 (0.81–2.17)	0.91 (0.54–1.57)	0.89 (0.59–2.24)	1.32 (0.91–1.32)	0.296
Testicular volume (ml)	1.60 (1.07–2.45)	2.00 (1.55–3.87)	1.40 (1.14–2.10)	1.29 (0.76–2.26)	1.32 (0.90–2.38)	0.036
GRI	0.72 (0.57–1.11)	0.58 (0.49–0.71)	0.70 (0.50–1.02)	0.94 (0.54–1.29)	0.97 (0.71–1.47)^a^	0.001
ALT (IU/L)	20 (14–30)	23 (16–32)	24 (17–38)	19 (14–26)	17 (11–22)	0.018
AST (IU/L)	20 (16–24)	19 (16–24)	21 (18–25)	20 (17–28)	19.00 (17–25)	0.374
ALP (IU/L)	99 (77–128)	68 (59–82)	89 (73–109)^a^	109 (92–122)^a^	143 (125–166)^a^	0.000
GGT (IU/L)	14 (11–18)	16 (12–19)	16 (12–24)	14 (11–18)	14.00 (11–15)	0.055
BUN (mmol/L)	4.98 ± 1.16	4.83 ± 1.16	5.19 ± 1.21	4.88 ± 0.98	5.05 ± 1.33	0.695
Cr (μmol/L)	61.43 ± 9.73	63.04 ± 8.94	63.22 ± 11.78	58.88 ± 9.62	60.95 ± 8.14	0.365
UA (μmol/L)	303 (255–354)	313 (274–364)	311 (266–381)	280 (234–338)	285 (255–346)	0.208
TG (mmol/L)	1.08 (0.72–1.67)	1.15 (0.67–1.62)	1.28 (0.71–2.01)	1.13 (0.79–2.00)	0.92 (0.66–1.17)	0.287
TC (mmol/L)	4.11 (3.52–4.59)	3.96 (3.50–4.56)	4.20 (3.62–4.67)	4.14 (3.67–4.86)	4.09 (3.23–4.41)	0.426
HDL-C (mmol/L)	1.31 (1.07–1.48)	1.12 (1.01–1.40)	1.33 (1.12–1.47)	1.37 (1.16–1.51)	1.35 (1.07–1.59)	0.129
LDL-C (mmol/L)	2.48 ± 0.71	2.48 ± 0.73	2.50 ± 0.68	2.60 ± 0.78	2.32 ± 0.63	0.618
FBG (mmol/L)	5.00 (4.80–5.22)	5.00 (4.83–5.18)	5.10 (4.76–5.25)	5.00 (4.80–5.30)	5.06 (4.86–5.23)	0.988
2hPG (mmol/L)	6.12 ± 1.18	6.54 ± 1.07	6.11 ± 1.21	6.07 ± 1.41	5.74 ± 0.86	0.148
25(OH)D (nmol/L)	35.73 (27.15–48.80)	32.22 (22.04–40.98)	41.40 (31.15–55.75)	41.36 (26.30–50.49)	33.30 (22.85–44.22)	0.204
PTH (pg/ml)	38.05 (30.49–48.83)	40.30 (29.98–47.73)	37.40 (28.64–57.73)	34.25 (29.98–46.40)	38.65 (32.33–49.08)	0.638

a *Significantly different from the first quartile in post-test. Bonferroni post-test analysis was used*.

### Serum OC Was Negatively Related to Testicular Volume and Positively Related to the Responsiveness of the Testicle to Gonadotropin in Male IHH Patients

**Figure 1:**
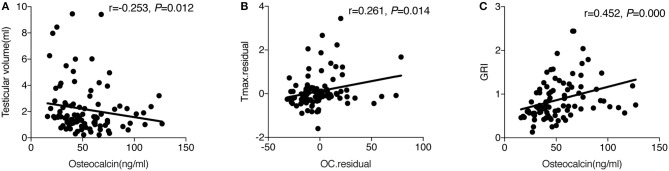
**(A)** The simple correlation between OC and testicular volume. **(B)** Partial correlation between OC and Tmax adjusted for age and testicular volume. The *y*-axis represents the residuals obtained after fitting a linear regression model using Tmax as a dependent variable, and age and testicular volume as the independent variables. The *x*-axis represents the residuals obtained after fitting a linear regression model using osteocalcin as a dependent variable, and age and testicular volume as the independent variables. **(C)** The simple correlation between OC and GRI.

Spearman correlation showed that OC was negatively related to testicular volume (*r* = −0.253, *P* = 0.012) (Figure 1A) and remained significant after adjusting for age, LH, TT, and 25(OH)D (*r* = −0.210, *P* = 0.047). No association was found between OC and LH, FSH, TT, FT, and Tmax in the simple correlation analysis. However, when age and testicular volume were adjusted, which were the two factors associated with OC (*r* = −0.607, *P* = 0.000) and Tmax (*r* = 0.744, *P* = 0.000), respectively, there was a positive association between OC and Tmax (*r* = 0.261, *P* = 0.014) (Figure 1B). When we further adjusted for other factors related to Tmax, such as LH and fat%, OC was still positively related to Tmax (*r* = 0.252, *P* = 0.045). Furthermore, OC was positively correlated with GRI (*r* = 0.452, *P* = 0.000) (Figure 1C), and such a correlation still tended to be significant after adjusting for age (*r* = 0.207, *P* = 0.053).

GRI is an index that incorporates Tmax and testicular volume, and we further explored the factor(s) responsible for the changes in GRI with multivariate stepwise regression analysis. It was demonstrated that OC was the determinant of GRI (adjusted *R*^2^ for the model = 0.093, *P* = 0.003) (Table 2).

**Table 2:** Factors determining the variation of GRI in multivariate stepwise regression analysis.

**Independent variables**	**Variables in final model**	**β**	**Standard error**	**Standardized β**	***P***
Age, OC, BMI, LH, FSH, TT, 25(OH)D, Cr	OC	0.006	0.002	0.322	0.003

### Serum OC Is a Determinant of Variations in Fat% and 2hPG in IHH Patients

As demonstrated in the differences between quartiles of OC, Spearman correlation analysis showed that serum OC was negatively related to BMI (*r* = −0.307, *P* = 0.002), body fat% (*r* = −0.415, *P* = 0.000), and 2hPG (*r* = −0.283, *P* = 0.005) but not to FBG. Since TT may influence the changes in metabolic parameters, including fat mass and blood glucose, in IHH patients (11), and age was associated with OC level in our study, we adjusted these two confounders and revealed that OC still had a negative correlation with fat% (*r* = −0.237, *P* = 0.047) and 2hPG (*r* = −0.274, *P* = 0.007) but not with BMI.

To further determine the contribution of OC in the changes in fat% and 2hPG, multivariate stepwise regression analysis was performed. The analysis showed that OC was the only determinant of fat% (adjusted *R*^2^ for the model = 0.089, *P* = 0.007) and 2hPG (adjusted *R*^2^ for the model = 0.047, *P* = 0.023) among other variables, such as age, BMI, TT, FT, LH, FSH, 25(OH)D, and Cr (Table 3).

**Table 3:** Factors determining the variations in body fat% and 2hPG in multivariate stepwise regression analysis.

**Dependent variables**	**Independent variables**	**Variables in final model**	**β**	**Standard error**	**Standardized β**	***P***
Fat%	Age, OC, TT, FT, LH, FSH, 25(OH)D, Cr	OC	−0.070	0.025	−0.319	0.007
2hPG	Age, OC, BMI, TT, FT, LH, FSH, 25(OH)D, Cr	OC	−0.012	0.005	−0.241	0.023

## Discussion

The major finding of the current study was that in male IHH patients, OC has an independent negative relationship with testicular volume and a positive association with Tmax; OC is also a major determinant of GRI, fat%, and 2hPG in IHH patients.

Mice lacking OC (OC^−/−^) exhibited decreased testicular volume and T level (16). In contrast to the expectation that OC might be positively associated with testicular volume in IHH patients, we found a negative association between these two markers. In determining the testicular volume, the germ cell population contributes, on average, for more than 60% in adult males. The correct tropism of the seminiferous tubule is provided by FSH, whose production is regulated also by inhibin B. However, we did not measure the sperm count in this group of patients because the testicles and genitals of most patients were too naive to meet the standard of semen examination because they were unable to masturbate. As for inhibin B, it regulates FSH in a negative feedback manner, but inhibin B level is low in both IHH and Klinefelter's syndrome patients (24). Thus, when FSH secretion is congenitally deficient, the regulation of FSH by inhibin B is probably inapplicable. In addition, OC level is not correlated to FSH in our study, so the relationship between OC and testis volume may be independent of FSH and inhibin B. This finding is worthy of discussion. In OC^−/−^ mice, the number of Leydig cells was not significantly affected by the absence of OC, nor was the expression of genes affecting Leydig cell proliferation (16), suggesting that the OC level may not always be positively linked with testicular volume, although the number of Leydig cells is not necessarily equal to the testicular volume. Moreover, the administration of OC to LH-deficient (Lhb^−/−^) mice did not lead to an increase in testicular volume or Leydig cell proliferation (25). This observation again indicates that OC may not have a direct stimulatory impact on or a positive association with testicular volume in the absence of LH. Notably, OC was significantly higher in both male Lhb^−/−^ mice and male IHH patients (9, 18). Thus, we hypothesized that these findings in mice and ours of the negative association between OC and testicular volume in IHH patients might be a compensatory mechanism to impaired testicular function. However, this hypothesis should be investigated in experimental and human studies. It was noticed that some studies reported that the basal testis volumes of IHH patients were as large as 3–6 ml (26). However, a study from China reported that the mean testis volume of IHH patients with a mean age of 23.6 years was 1.7 (0.9–3.4) ml for Group A and 1.5 (1.0–3.0) ml for Group B (27), and another study from Turkey showed that the mean testicular volume of IHH patients was 1.4 ± 1.5 ml (28), which is comparable to our data.

In the murine model, OC promotes testosterone biosynthesis (16), but the results are inconsistent among clinical studies. Studies from male patients with obesity, type 2 diabetes, or hyperthyroidism have shown that serum OC was positively associated with testosterone (29–31). However, in young male adults from infertile couples, OC did not correlate with testosterone after adjusting for age and BMI (32). The different underlying pathophysiological abnormalities might be responsible for such differences regarding the associations between OC and TT or FT. Therefore, the reason why there was no positive correlation between OC and baseline TT or FT in our study might be the absence of LH in IHH patients. It should strengthen the proper endocrine milieu during puberty, as a major factor in testis development and function, which may also influence the response to steroidogenic factors in adult life.

Unlike the results in Lhb^−/−^ mice, despite a high level of LH in OC^−/−^ mice, injection of LH into OC^−/−^ mice normalized the testosterone level and led to an increase in testicular volume (25), which means that when lacking OC, more LH is needed to trigger testosterone biosynthesis and development of the testicle. This, in turn, indicates that OC may act as an “enhancer” to facilitate the responsiveness of testicle to produce testosterone upon gonadotropin stimulation in IHH. This hypothesis seems to be supported by our study. The response of serum TT to gonadotropin stimulation is commonly employed to evaluate testicular function in human studies (3, 33–35). Thus, in this study, we used Tmax to represent the maximal responsiveness of the testicle and to explore its relationships with other related parameters. We found that OC was independently and positively related to Tmax and was responsible for the changes in GRI in IHH patients, suggesting that OC might play a role in testosterone secretion when it receives gonadotropin stimulation, such as in conditions of IHH. However, GRI is a novel measure proposed in this article for the first time, which needs to be independently verified in future studies.

In addition to the effect on male reproductive function, OC is also actively involved in the modulation of energy metabolism (19–21). Cross-sectional and some longitudinal studies revealed that higher serum levels of OC are associated with lower plasma glucose levels, improved glucose tolerance, β-cell function, and insulin sensitivity in individuals with different ages and glucose metabolism status (36). Higher circulating OC levels are also correlated with less total and visceral fat and are positively associated with fat-free mass in premenopausal women (37). IHH patients often suffer a variety degrees of metabolic dysfunction, such as elevated FBG, HOMA-IR, and fat% (6–8). Likewise, we found that OC is negatively associated with fat% and 2hPG after adjusting for age and TT. This finding indicated that the metabolic function of OC on glucose and fat mass also exists in IHH.

Our study has several limitations. First, other bone turnover markers and the undercarboxylated OC (ucOC), which was found to be the metabolic active form of OC in mouse studies, were not measured; hence, the findings could be representative of bone turnover in general and it is not clear whether the absence of correlation between OC and TT or FT in this study was caused by not measuring the ucOC. However, serum concentrations of ucOC and total OC are highly correlated in humans (38). Second, as IHH patients had extremely low baseline TT and FT levels, which were often below or around the minimum level of measurement range, it is difficult to reveal the precise relationship between TT and FT with other parameters. In addition, fat% by bioimpedance is not always accurate. Third, this is a cross-sectional study and the correlations were significant but weak, and the results may be biased by the sample size. However, considering the low incidence of IHH and the largest number of cases reported in single center studies was 215 (39), the results derived from our study are still informative. Additional mouse studies and large clinical prospective investigations are required to explore the ability of OC to favor gonadotropin responsiveness in patients with hypogonadism, including IHH.

In conclusion, the current study revealed that OC is independently and positively related to the responsiveness of the testicle to gonadotropin in IHH male patients. Whether this observation can translate into the clinical implication that a higher OC is indicative of a better therapeutic efficacy in IHH patients receiving gonadotropin is worthy of further testing and verification.

## Data Availability Statement

The raw data supporting the conclusions of this manuscript will be made available by the authors, without undue reservation, to any qualified researcher.

## Ethics Statement

The studies involving human participants were reviewed and approved by Institutional Review Board of the Rui-jin Hospital. The patients/participants provided their written informed consent to participate in this study.

## Author Contributions

S-YS and J-ML were the guarantors of this work and, as such, had full access to all the data in the study and take responsibility for the integrity of the data and the accuracy of the data analysis and involved in the conception and design of the study. Y-YY and S-CZ collected and analyzed the data. W-QW, Z-WY, Y-WZ, YQ, Y-HC, W-QG, W-CW, and H-YZ were involved in the collection and interpretation of data. Y-YY drafted the manuscript. CS modified the manuscript. J-ML revised the manuscript for important intellectual content. All authors gave final approval of the version to be published.

### Conflict of Interest

The authors declare that the research was conducted in the absence of any commercial or financial relationships that could be construed as a potential conflict of interest.
